# One health approach to toxocariasis in Brazilian indigenous populations, their dogs, and soil contamination

**DOI:** 10.3389/fpubh.2023.1220001

**Published:** 2023-09-07

**Authors:** Vamilton Alvares Santarém, Fernando Rodrigo Doline, Isabella Braghin Ferreira, João Henrique Farinhas, Leandro Meneguelli Biondo, Roberto Teixeira de Souza Filho, Christina Pettan-Brewer, Rogério Giuffrida, Susana Angélica Zevallos Lescano, Andrea Pires dos Santos, Louise Bach Kmetiuk, Alexander Welker Biondo

**Affiliations:** ^1^Graduate College in Animal Sciences, University of Western São Paulo (UNOESTE), Presidente Prudente, São Paulo, Brazil; ^2^Graduate College of Cell and Molecular Biology, Federal University of Paraná (UFPR), Curitiba, Paraná, Brazil; ^3^National Institute of the Atlantic Forest (INMA), Brazilian Ministry of Science, Technology, and Innovation, Santa Teresa, Espirito Santo, Brazil; ^4^Department of Comparative Medicine, School of Medicine, University of Washington, Seattle, WA, United States; ^5^Laboratory of Medical Investigation, Institute of Tropical Medicine of São Paulo, University of São Paulo, São Paulo, Brazil; ^6^Department of Comparative Pathobiology, Purdue University, West Lafayette, IN, United States

**Keywords:** traditional populations, epidemiology, *Toxocara* spp., risk factors, zoonosis

## Abstract

**Introduction:**

Although socioeconomic vulnerability and lifestyle factors may contribute to the transmission of *Toxocara* spp., no study has investigated indigenous populations in Brazil using the One Health approach.

**Methods:**

Accordingly, this study assessed anti-*Toxocara* spp. antibodies in Brazilian indigenous people and healthcare professionals by enzyme-linked immunosorbent assay. Presence of *Toxocara* spp. eggs (feces and hair) in dogs as definitive hosts and in soil samples of the indigenous communities were also recovered and molecularly investigated.

**Results:**

Overall, 342/463 (73.9%) indigenous individuals and 46/147 (31.3%) non-indigenous healthcare professionals were seropositive for *Toxocara* spp. In addition, *T. canis* eggs were retrieved from 9/194 (4.6%) dog fecal samples and 4/204 (2.0%) dog hair samples, mainly from the Paraná State communities (3/42; 7.1%). Soil contamination was observed only in the Paraná State communities (36/90; 40.0%), with the molecular detection of *T. canis*. River water consumption was also associated with indigenous seropositivity (Odds ratio, 11.4).

**Discussion:**

Indigenous individuals in Paraná State communities were 2.72-fold more likely to be seropositive than those in São Paulo State, likely due to a lack of sanitary infrastructure. In this scenario, a primarily soil-transmitted disease may also have become waterborne, with embryonated eggs probably spread to water supplies by rain. Full-time healthcare professionals in daily contact with indigenous communities were 9.2-fold more likely to be seropositive than professionals who visited sporadically, suggesting exposure to *Toxocara* spp. during their work and raising health concerns. In addition, the findings herein showed a significantly higher seroprevalence in indigenous people than in healthcare workers (*χ*^2^ = 85.5; *p* < 0.0001), likely due to overtime exposure to *Toxocara* spp. In conclusion, Brazilian indigenous communities are highly exposed to toxocariasis, with poor infrastructure and contact with contaminated river water as associated risk factors and a higher risk of infection in healthcare professionals working full-time in these communities.

## 1. Introduction

The indigenous population in Brazil has been estimated in approximately 900,000 individuals, mostly living in rural areas (63.8%). The indigenous population represents approximately 0.4% of the Brazilian general population (1). Lower socioeconomic and educational levels and ethnic lifestyle behaviors may have historically exposed indigenous communities to zoonotic pathogens (2), including *Toxocara* spp., helminths mainly harbored by dogs (*T. canis*) and cats (*T. cati*) (3).

Adult female *Toxocara* spp. may release several hundred thousand non-embryonated eggs per day after reproducing in the small intestine of their definitive host, which may be released into the environment via feces and embryonate over a period of 2 to 6 weeks (3). *Toxocara* spp. can develop and tolerate a wide range of ambient temperatures (4) surviving under moist and temperature conditions for as long as 2 to 4 years or more (5). *Toxocara* spp. eggs are mostly shed by dogs and kittens due to the high number of larvae transmitted by transplacental and lactogenic via, respectively (6).

Severity of toxocariasis in the definitive hosts depends on the burden of adult worms colonizing the small intestine. The adult forms compete with the host for nutrients, and cause enteritis which induces absorption decrease, vomiting, intestinal occlusion, and even death in massive infection. In puppies, larval migration through the organs may cause cough, rhinorrhea, and seizure (6). Kittens infected with high burdens may commonly present with poor body condition, pot-bellied appearance, respiratory disorders, diarrhea, vomiting, and cachexia in extreme cases (7). With age (6 months to 1 year), the larvae tend to become developmentally arrested in tissues of dogs and cats (8).

Visceral toxocariasis (or visceral larva migrans) may induce mainly hepatic (9, 10) pulmonary disorders (11, 12), while ocular form may cause visual impairment and blindness (13–16). Neurological form (neurotoxocariasis) may cause disturbances in the central nervous system, including cerebral vasculitis, meningitis, meningoencephalitis, seizures, and cognitive impairment (5, 17, 18). Cutaneous manifestations (urticaria, pruritus, erythematous rush) accompanied by eosinophilia have been associated with toxocariasis (19, 20). For instance, a case of eosinophilic panniculitis (EP) in a 5-year-old Brazilian girl was associated to toxocariasis (21).

Seroprevalence of toxocariasis has been estimated in 19.0% worldwide, and in 27.6% in Brazil according to a metanalytic study (22). In indigenous populations, seroprevalence of *Toxocara* spp. worldwide has ranged between 4.8% (9/188) in Malaysian Orang Asli individuals and 76.6% (252/329) in aboriginal school children of Taiwan (23). Indigenous communities may be more exposed to toxocariasis, as contact with soil, drinking river water and agriculture activities have been associated with high seroprevalence [383/483; 79.3%; 95% (CI): 75.5–82.3] in Colombian indigenous individuals (24). Not surprisingly, the highest toxocariasis seroprevalence in Brazil were observed in rural conditions, including 503/791 (63.6%) school-age children of a small town of northeastern (25) and in 247/344 (71.8%) adult inhabitants of rural southern Brazil (26).

One Health approach has been considered highly applicable for indigenous communities due to their intimate relationship with the environment, particularly linked to their heritage beliefs and health understandings (2). While lifestyle factors and socioeconomic vulnerabilities in Brazil may contribute to *Toxocara* spp. circulation among human, animals and environment in indigenous populations, no study to date has investigated their impact on indigenous health, particularly using the One Health approach. Thus, this study aimed to assess *Toxocara* spp. seropositivity in indigenous people and healthcare professionals and the presence of *Toxocara* spp. eggs in dogs and soil samples from nine Brazilian indigenous communities.

## 2. Materials and methods

### 2.1. Ethics statement

This study initially received approval from three different indigenous instances, which were later submitted together and approved by the Committee on Ethics in Human Health of the Brazilian Ministry of Health (protocol 52039021.9.0000.0102) and by the Committee on Ethics in the Use of Animals (protocol number 033/2021) at the Federal University of Paraná.

### 2.2. Study design

This study used a cross-sectional seroepidemiological One Health approach to examine the seroprevalence of toxocariasis in indigenous communities of Paraná (southern Brazil) and São Paulo (southeastern Brazil). Both indigenous people and healthcare professionals were assessed for *Toxocara* spp. seroprevalence and associated risk factors. In addition, *Toxocara* spp. eggs were analyzed in dog hair, feces, and soil samples.

### 2.3. Study area

Indigenous participants from Guarani, Terena, and Kaingang communities were also sampled. The communities were located in the states of Paraná and São Paulo, and sampling was conducted from December 2020 to February 2022. The geographic locations and total populations sampled from indigenous communities are presented in Supplementary Table S1.

### 2.4. Population characteristics

#### 2.4.1. Indigenous communities

The socioeconomic characteristics of indigenous communities are distinct in the states of Paraná and São Paulo (Supplementary Figures S1–S5). The indigenous population living in the communities of Paraná State has strong environmental ties and relies on natural resources for sustenance, such as wildlife hunting, fishing, and small subsistence agriculture (27). Craftsmanship using natural resources is a form of secondary income and includes making baskets, necklaces, miniature wildlife animals carved in wood, spears, bows, and arrows (27, 28). These communities lack water treatment systems and septic tanks in their households. The indigenous communities of São Paulo rely on agriculture as their main economic activity (outside trading) and subsistence (29). Individuals in these indigenous communities also work in nearby rural farms and urban areas with low hunting activity (30). Furthermore, handicrafts are a source of income for families in these communities (31). The indigenous communities of São Paulo have artesian wells for water supply and septic tanks for feces disposal.

According to the Special Secretaria for Indigenous Health (SSIH) administration, indigenous populations undergo a preventive deworming program with commercially available active pharmaceutical ingredients (albendazole) twice a year (May and November) to control the risk of helminth infection.

#### 2.4.2. Healthcare professionals

The SSIH was established in 2010 by the Brazilian Ministry of Health to improve indigenous health and employs 22,000 healthcare professionals (52.0% indigenous) who provide local healthcare services to an estimated indigenous population of 900,000 individuals (46.4% under 19 years of age), representing approximately 0.4% of the total Brazilian population (1).

In addition to indigenous populations, non-indigenous healthcare professionals were also sampled during incursions and specific visits to the Special Department of Indigenous Health (SDIH), Seashore South. The Seashore South SDIH was one of 34 national divisions under the SSIH of the Brazilian Ministry of Health at the time and managed more than 25,000 indigenous individuals from 25 ethnic groups living in an area of 174,521.07 km^2^ (43.13 million acres) with 129 indigenous communities located in four Brazilian states (Santa Catarina, Paraná, São Paulo, and Rio de Janeiro) (32).

Healthcare professionals were classified into groups according to their level of contact, frequency of visits to indigenous populations, and function. The first group (high-level contact) consisted of professionals such as physicians, nurses, nursing technicians, drivers, and teachers who frequently visited indigenous communities (5 days per week). The second group (medium-level contact) included multidisciplinary SDIH professionals who visited the communities regularly (one to two times/month). The third group (low-level contact) included administrative and healthcare professionals who visited the indigenous communities sporadically (one to two times/year).

### 2.5. Sample collection

#### 2.5.1. Human blood samplings

Human participants, including indigenous people and non-indigenous healthcare professionals, were sampled after signing a consent form and completing an epidemiological questionnaire. Certified nurses collected blood samples (10 mL) via cephalic venipuncture. Whole blood samples were placed in sterile vacuum tubes containing a serum separator gel without an anticoagulant. Blood samples were kept at room temperature (25°C) until visible clots formed and were centrifuged at 800 g for 5 min. The serum samples were kept at −20°C until use.

#### 2.5.2. Dog feces and hair samplings

The dog fecal samples were collected from the rectum and stored individually in graduated tubes (50 mL) containing 10% formalin solution, followed by refrigeration at 4°C until microscopic examination (8). Dog hair samples were collected from the perineal and lower back regions using sterile scalpel blades, placed in individual graduated tubes (50 mL), and kept under refrigeration (4°C) until processing.

#### 2.5.3. Soil samplings

A total of 90 soil samples were collected in Paraná (30 samples per community) and 40 in São Paulo (10 samples per community) states. Soil samples were randomly collected in common areas of each indigenous community and the number of sets was ruled by the presence of soil surround each area. Further, sets presenting grass or feces were not sampled. The soil sampling sets and the number of samples collected per community is presented in Supplementary Table S2.

Approximately 50 g of soil were collected at 5 to 15 cm depth, placed in individual plastic bags, and refrigerated at 4°C until testing, following protocol described elsewhere (33).

### 2.6. Human serological tests

#### 2.6.1. *Toxocara canis* excretory-secretory (TES) antigen preparation

Adult *T. canis* nematodes were previously collected from naturally infected pups that spontaneously released the parasites. Female nematodes were treated with 1% sodium hypochlorite for 5 min and washed with 0.9% saline for 3 min to remove debris. Eggs were collected by sectioning the anterior third worm body segment and incubating in 2% formalin at 28°C for approximately 30 days. The larvae hatched from the eggs were incubated (37°C) in serum-free Eagle medium, following a previously described protocol (34). Weekly, the culture supernatant was removed and treated with 5.0 μL/mL of the protease inhibitor phenylmethylsulphonyl fluoride (PMSF; 200 mM), concentrated with a commercially available kit (Amicon Ultra Centrifugal Filter Unit, Millipore, Danvers, MA, USA), dialyzed with distilled water, centrifuged (18,500 g for 60 min at 4°C) and filtered using 0.22 μm filter membranes (Millipore). The protein concentration was determined using a previously described method (35).

#### 2.6.2. Pre-adsorption of sera with *Ascaris suum* adult worm extract

The specificity of the enzyme-linked immunosorbent assay (ELISA) was improved by pre-adsorbing serum samples with *A. suum* adult worm extracts (AWE) using a previously established protocol, eliminating antibodies elicited by exposure to *Ascaris* spp., which could cause cross-reactivity with *Toxocara* spp. antigens (36). Adult nematodes were recovered from the intestines of slaughtered pigs and macerated in distilled water. Next, one part of NaOH (1.5 M) was added to nine parts of water, and the mixture (final concentration of 0.15 M) was incubated at room temperature for 2 h. The pH of the mixture was neutralized with 6 M HCI and centrifuged at 18,500 g for 20 min at 4°C. The lipids were removed with ether, and the supernatant was filtered through 0.22 μm filter membranes. All serum samples were pre-incubated with an AWE solution (25.0 μg/μL) in 0.01 M phosphate-buffered saline (PBS, pH 7.2) containing 0.05% Tween 20 (PBS-T) (Sigma, St. Louis, MO, United States) for 30 min at 37°C.

#### 2.6.3. Indirect ELISA

ELISA was performed using polystyrene 96-well microtiter plates (Corning, Costar, New York, USA) coated with TES antigens (1.9 μg/μL per well) in 0.06 M carbonate–bicarbonate buffer at a pH of 9.6 for 1 h at 37°C and 18 h at 4°C. Plates were blocked with 3% commercial skim milk in 5% PBS-Tween for 1 h at 37°C. The serum samples were previously adsorbed with *A. suum* somatic antigen (AWA) diluted 1/80 in microtubes with PBS-Tween-Milk (25 μg/mL), incubated for 30 min at 37°C, then diluted (1/200) in plates with PBS-T-Milk 5%, incubated for 1 h at 37°C, washed three times for 5 min each (in duplicate). After this stage, plates were incubated with anti-human immunoglobulin G (IgG) (Fc-specific) peroxidase antibody produced in goats (Sigma A6029) at a 1:5000 dilution (45 min at 37°C), followed by three 5 min washes. The reaction was performed using an o-phenylenediamine substrate (0.4 mg/mL, Sigma), and 2 N H_2_SO_4_ sulfuric acid was added to stop the reaction.

Positive and negative controls were included in each plate. The absorbance was read at 492 nm, and the cutoff value was defined as the mean absorbance of 90 negative control sera plus three standard deviations. This assay exhibited 78.3% sensitivity and 92.3% specificity, as previously reported (37, 38). Antibody levels were calculated as the ratio between the absorbance values of each sample and the cutoff value, expressed as reactivity indices set at 0.400.

All the samples were tested at the Laboratory of Medical Investigation, Institute of Tropical Medicine of São Paulo, University of São Paulo, Brazil. Negative serum samples used as controls herein have been maintained in the serum bank and routinely used for serodiagnosis of toxocariasis by ELISA, and previously tested by an established protocol (34), ensuring that samples were negative for parasites in previous studies (39, 40). Accordingly, the cut-off value obtained for the IgG antibody corresponded to an optical density (OD) was of ≤0.5, considered as an absence of infection or a negative result (34, 41).

### 2.7. *Toxocara* spp. eggs recovery from feces and dog hair samples

Dog fecal samples were processed using a flotation technique in a hypersaturated sodium chloride (NaCl) solution (3). The solution (*d* = 1.20 g/cm^3^) was added to the samples, filtered through gauze, kept at rest (5 min), and examined under an optical microscope (objective: 10x) to identify parasitic structures.

The dog hair samples were processed according to a previously described protocol with some modifications (42). Distilled water (20 mL) and anionic detergent Tween 80 (5%, 0.2 mL) were added to the samples, homogenized by shaking, and incubated overnight. Then, the samples were homogenized in identical amounts of distilled water and detergent. The material was filtered through metal mesh sieves (300, 212, and 38 μm) in running water (5 min). The washing material (38 μm sieve) was centrifuged and analyzed microscopically (magnifications: 10x and 40x).

### 2.8. *Toxocara* spp. eggs recovery from soil samples

Soil samples were processed following previously described protocols, with some modifications (33, 43). For each sample, 20 g of soil was weighed and allowed to stand for 12 h with the anionic detergent Tween-80 5% (100 mL). Next, the supernatant was discarded, and Tween-80 was added (100 mL). The soil samples were filtered through metal mesh sieves (300, 212, 90, and 38 μm) in running water. The soil obtained in the last sieve (38 μm) was processed using a centrifugal flotation technique with a zinc sulfate solution (*d* = 1.35 g/cm^3^). The supernatant was transferred to a centrifuge tube, homogenized with distilled water (enough quantity to 15 mL), and centrifuged (2,500 rpm for 5 min). The supernatant was discarded, and the previous processes (distilled water addition, centrifugation, and supernatant disposal) were repeated three times to remove the zinc sulfate solution. Next, the pellet was microscopically analyzed (magnifications: 10x and 40x).

*Toxocara* spp. eggs recovered from the soil were classified into four groups: non-viable (wall-disrupted or not intact), viable (intact eggs with contents), embryonated eggs (with cellular division), or embryonated eggs (containing larva) (44). The eggs were collected and stored (−20°C) in microtubes with distilled water for molecular characterization.

### 2.9. DNA extraction and polymerase chain reaction (PCR) assay

#### 2.9.1. DNA extraction from eggs

Genomic DNA was extracted from the pool of *Toxocara* spp. eggs recovered from the soil of each community using a commercial purification kit (PureLink ™ Microbiome DNA purification kit; Invitrogen, Waltham, MA, United States), following the manufacturer’s instructions, with some modifications. The eggs were disrupted by homogenization (D160 Homogenizer, Scilogex, Rocky Hill, CT, United States) in a microtube containing a lysis buffer solution, followed by incubation with proteinase K solution (20 μL) at 65°C for 16 h. Genomic DNA for positive control samples was obtained from eggs produced by female *T. canis* and *T. cati* parasites in naturally infected dogs and cats. The DNA concentration was determined by measuring the absorbance at 260/280 nm using a spectrophotometer (NanoDrop 2000, Thermo Scientific, Waltham, MA, United States).

#### 2.9.2. PCR assay

The ribosomal DNA (rDNA) region, comprising partial sequences of ITS1 and ITS2, was analyzed molecularly using the primers previously described (45): *T. canis* (Forward:5′-CTC GAG TCG ACG AAG TAT GTA C-3′; Reverse:5′-AAT TGG GCC GCC CAT CAT AC-3′), and *T. cati* (Forward:5′-GTA AGA TCG TGG CAC GCG TAC GTA-3′; Reverse:5′-TCT TTG ATG TCA AGA CTT CAG CGC-3′). The reaction mixtures (volume of 25 μL) contained 10 μM of forward and reverse primers, 0.02 mM of deoxynucleotide, 30 mM of MgCl_2_, 2 μL of buffer (10x PCR Buffer), 1 U of Taq DNA polymerase (Invitrogen), and approximately 50 ng of DNA template. Amplifications were performed using a thermal cycler (Multigene, Labnet International, Edison, NJ, United States) under the following conditions: 94°C for 60 s, followed by 35 cycles at 94°C for 60 s, 55°C for 45 s and 72°C for 30 s, and a final cycle at 72°C for 5 min. The electrophoresis (60 min at 80 V) was carried out with the application of the PCR products (25 μL) with loading buffer 5x (4 μL) on a 1.5% agarose gel stained with ethidium bromide along with DNA ladder (100 bp). Amplification products were visualized under ultraviolet light.

### 2.10. Epidemiological data collection

During blood sampling, an epidemiological assessment was conducted using individual questionnaires with the assistance of an indigenous translator when necessary. The questionnaire assessed potential exposure to *Toxocara* spp. and included information regarding gender, age, ethnicity, educational level, community and time living in the community, occupation, hunting habits and frequency, owned animals, drinking water sources, habit of washing fruit and vegetables, washing hands before meals, and consumption of raw or undercooked meat. Participants who owned dogs could also sign up for animal sampling and respond to a questionnaire to gather information on their dog’s age, gender, breed, origin (purchased, given, or adopted), place of residence, diet, consumption of raw meat, drinking water source, access to the forest, hunting habits, ectoparasite and endoparasite presence and control, and vaccination.

To ensure veterinary intervention and ethical research, all sampled (and non-sampled voluntary brought) dogs were examined, and were given a rabies vaccine (Immunovet R, Biovet, São Paulo, SP, Brazil) and species-specific vaccine against distemper, hepatitis, adenovirus, parainfluenza, parvovirus, coronavirus, *Leptospira* serovars Canicola, Icterohaemorrhagiae, Copenhageni and Grippotyphosa (Poly 10, Lema-Injex Biologic, Lagoa Santa, MG, Brazil), oral antiworm treatment (Pyrantel Pamoate associated with Praziquantel), pour-on treatment for ticks and fleas (Fipronil), and dog treatment for sarcoptic mange and tungiasis (Ivermectin) were administered according to manufacturer’s recommendation. All products were purchased from certified veterinary drugstores with long expiration dates, and the vaccines were stored on recyclable ice until application. The owners were provided with a cell phone contact in case of dog immunoreaction or side effects. Additionally, vinyl banners against dog and cat abandonment were printed using some of our research funding and posted at the entry of each of the 96 indigenous communities located in Paraná State.

### 2.11. Statistical analysis

The association between potential risk factors and seropositivity for *Toxocara* spp. in indigenous people and healthcare professionals was tested by univariate analysis using the chi-square test or Fisher’s exact test, as appropriate. Predictor variables with statistical significance lower than 0.20 in the univariate analysis were subjected to multivariate logistic regression analysis. The odds ratios (ORs) with 95%CIs were calculated, and a value of p lower than 0.05 was considered statistically significant. The accuracy of the logistic model for predicting seropositivity in indigenous people and healthcare professionals was evaluated by estimating the area under the receiver operating characteristic (ROC) curve (AUC). All analyses were performed using the R program v. 4.2.2 (46).

## 3. Results

A total of 463 indigenous participants were sampled from nine indigenous communities of the Guarani, Terena, and Kaingang ethnic groups, 162 individuals from five communities located in Paraná State, and 301 individuals from four communities of São Paulo State. In addition to the indigenous populations, 147 non-indigenous healthcare professionals were sampled while they visited the SDIH-Seashore South. A total of 194 fecal and 204 hair samples were collected and analyzed from dogs in the indigenous communities. A total of 130 soil samples were collected, of which 90 were from Paraná, and 40 were from São Paulo.

**Figure 1:**
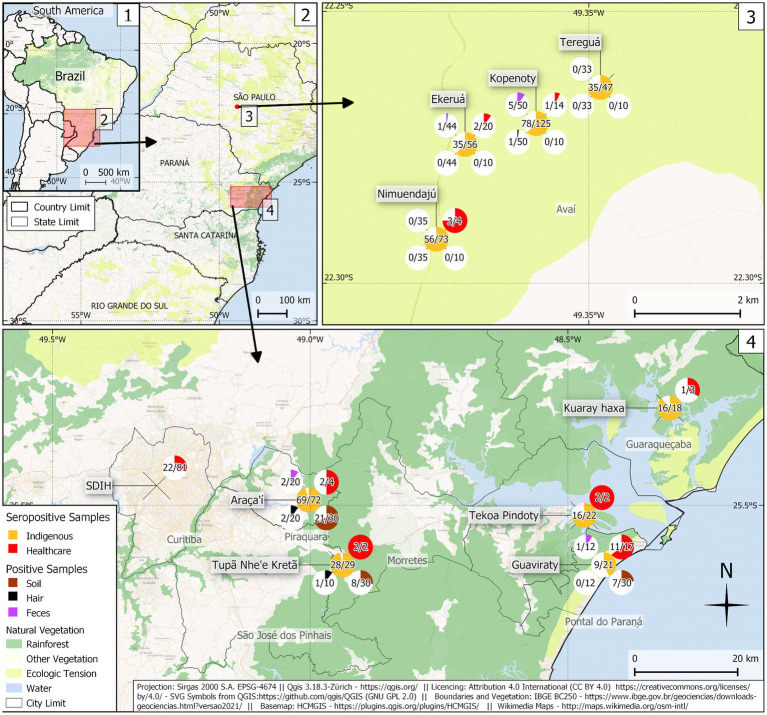
Sampling location, frequency of anti-*Toxocara* spp. antibodies in indigenous individuals and healthcare professionals, and the presence of feces, hair, and soil-positive samples in indigenous communities of Paraná and São Paulo States. (1) Map of Brazil; (2) Map of Paraná and São Paulo States; (3) Map of indigenous communities in São Paulo State (southeastern Brazil); (4) Map of indigenous communities in Paraná State (southern Brazil).

Overall, anti-*Toxocara* IgG antibodies were detected in 342/463 (73.9%; 95%CI:70.0–77.7) indigenous individuals living in Paraná State associated with a 2.72-fold (95%CI,1.7–4.4) more likelihood of seropositivity, when compared to São Paulo State. In addition, 46/147 (31.3, 95%CI:24.4–39.2) non-indigenous healthcare professionals were seropositive, most of whom worked in the indigenous communities of Paraná State. The chi-square test showed that the seroprevalence in indigenous individuals was statistically higher (*χ*^2^ = 85.53; df = 1; *p* < 0.0001) than in non-indigenous healthcare professionals (Figure 1, Table 1).

**Table 1:** Prevalence rates (%) for anti-*Toxocara* IgG antibodies in indigenous populations (*N* = 463) living in nine communities of Paraná and São Paulo States, Brazil.

Indigenous communities	Positive ELISA	Participants	Prevalence (%) (95%CI)
Paraná State – South			
Tekoa Pindoty	16	22	72.7 (51.9–86.9)
Kuaray haxa	16	18	88.9 (67.2–96.9)
Araça’í	69	72	95.8 (88.5–98.6)
Tupã Nhe’e Kretã	28	29	96.5 (82.8–99.4)
Guaviraty	9	21	42.9 (24.5–63.5)
Subtotal	138	162	85.2 (78.9–89.8)
São Paulo State – Southeast			
Kopenoty	78	125	62.4 (53.7–70.4)
Tereguá	35	47	74.5 (60.5–84.8)
Ekeruá	35	56	62.5 (49.4–74.0)
Nimuendajú	56	73	76.7 (65.8–84.9)
Subtotal	204	301	67.8 (62.3–72.8)
Total	342	463	73.9 (69.7–77.7)

### 3.1. Associated risk factors to human *Toxocara* spp. seropositivity

The risk factors associated with *Toxocara* spp. seropositivity in indigenous people were analyzed and are presented (Table 2). The final logistic regression model revealed that using river water as the water source was the only predictive factor for human toxocariasis, with an 11.4-fold (95%CI,4.6–37.8) increased risk of seropositivity compared to artesian wells. Although the univariate analysis included gender in the logistic regression (*p* = 0.1), this variable was not considered statistically significant in the final model (*p* = 0.155). Other risk factors were not statistically significant in the univariate analysis, including age (*p* = 0.542), educational level (*p* = 0.355), consumption of raw/undercooked meat (*p* = 0.558), game meat consumption (*p* = 0.547), cat ownership (*p* = 0.686), and dog ownership (*p* = 0.349). The performance of the regression final model according to the ROC curve (AUC:65.3%; 95%CI:60.6–70.0) was considered fair (Supplementary Figure S6).

**Table 2:** Associated risk factors to anti-*Toxocara* (IgG) antibodies seropositivity in indigenous persons (*N* = 463) in Paraná and São Paulo States, Brazil, by uni and multivariate analyses.

			Univariate analysis		Multivariate analysis		Seropositive (%)	Seronegative (%)	OR (95%CI)	*p* value	OR (95%CI)	*p* value
**Characteristic**	342 (73.9)	121 (26.1)				
**Gender**				0.1		
Female	169 (49.4)	71 (58.7)	1.0 [Reference]		1.0 [Reference]	
Male	173 (50.6)	50 (41.3)	1.45 (0.96–2.22)		1.38 (0.89–2.12)	0.155
**Age (years old)**				0.542		
03 to 17	104 (30.4)	33 (27.3)	1.0 [Reference]			
18 to 26	76 (22.2)	22 (18.2)	1.09 (0.59–2.05)			
27 to 40	84 (24.6)	32 (26.4)	0.83 (0.47–1.47)			
41 to 89	78 (22.8)	34 (28.1)	0.73 (0.41–1.28)			
**Educational level**				0.244		
Illiterate	9 (2.6)	4 (3.3)	1.0 [Reference]			
Elementary	173 (50.6)	73 (60.3)	1.07 (0.27–3.48)			
High School	127 (37.1)	34 (28.1)	1.69 (0.42–5.64)			
College	33 (9.7)	10 (8.3)	1.47 (0.33–5.81)			
**Drinking river water**				<0.001		
No	245 (71.6)	117 (96.7)	1.0 [Reference]		1.0 [Reference]	
Yes	97 (28.4)	4 (3.31)	11.1 (4.51–37.8)		11.4 (4.61–37.81)	<0.0001
**Consumption of raw meat**				0.558		
No	326 (95.3)	113 (93.4)	1.0 [Reference]			
Yes	16 (4.7)	8 (6.6)	0.69 (0.29–1.76)			
**Consumption of game meat**				0.547		
No	154 (45.0)	59 (48.8)	1.0 [Reference]			
Yes	188 (55.0)	62 (51.2)	1.16 (0.77–1.76)			
**Owning cat**				0.686		
No	183 (53.5)	68 (56.2)	1.0 [Reference]			
Yes	159 (46.5)	53 (43.8)	1.11 (0.73–1.70)			
**Owning dog**				0.349		
No	75 (21.9)	21 (17.4)	1.0 [Reference]			
Yes	267 (78.1)	100 (82.6)	0.75 (0.43–1.27)			

In the final logistic regression analysis of non-indigenous healthcare professionals, higher seropositivity was found in males (OR:2.3; 95%CI =1.0–5,1; *p* = 0.048) compared to females and daily work (high-level contact group) (OR:9.2; CI 95% = 2.3–49.9; *p* = 0.004) compared to sporadic visits to indigenous communities (low-level contact group) (Table 3). A seropositivity tendency was observed with increasing age (*p* = 0.002) in the univariate analysis but was not confirmed by logistic regression. The consumption of water provided by indigenous communities (*p* = 0.289) and the ingestion of raw or undercooked meat (*p* = 1.0) were not statistically significant. The ROC curve of the logistic model for non-indigenous healthcare professionals (AUC:78%) (95%CI,70.3–85.8) showed satisfactory to good performance (Supplementary Figure S7).

**Table 3:** Associated risk factors to anti-*Toxocara* (IgG) antibodies seropositivity in healthcare professionals (*N* = 147) of Paraná and São Paulo States, Brazil, by uni and multivariate analyses.

	ELISA result		Univariate analysis		Multivariate analysis		Seropositive (%)	Seronegative (%)	OR (95%CI)	*p* value	OR (95%CI)	*p* value
**Characteristic**	46 (31.3)	101 (68.1)				
**Gender**				0.111		
Female	22 (47.8)	64 (63.4)	1.0 [Reference]		1.0 [Reference]	
Male	24 (52.2)	37 (36.6)	1.88 (0.92–3.84)		2.25 (1.02–5.13)	0.048
**Age (years old)**				0.002		
20 to 29	6 (13.0)	36 (35.6)	1.0 [Reference]			
30 to 38	7 (15.2)	26 (25.7)	1.60 (0.47–5.65)		0.92 (0.23–3.74)	0.912
39 to 46	18 (39.1)	18 (17.8)	5.77 (2.02–18.6)		3.02 (0.84–11.7)	0.096
47 to 65	15 (32.6)	21 (20.8)	4.15 (1.44–13.4)		2.58 (0.74–9.67)	0.143
Working regimen in communities				<0.001		
Sporadically (1–2 times/year)	3 (6.52)	32 (31.7)	1.0 [Reference]		1.0 [Reference]	
Periodically (1–2 times/month)	20 (43.5)	51 (50.5)	3.98 (1.22–18.7)		3.01 (0.78–15.16)	0.135
Frequently (5 days per week)	23 (50.0)	18 (17.8)	12.7 (3.72–61.8)		9.22 (2.27–49.28)	0.004
Consumption of water in communities				0.289		
No	31 (67.4)	78 (77.2)	1.0 [Reference]			
Yes	15 (32.6)	23 (22.8)	1.64 (0.74–3.55)			
Ingestion of raw meat				1.0		
No	41 (89.1)	89 (88.1)	1.0 [Reference]			
Yes	5 (10.9)	12 (11.9)	0.92 (0.27–2.70)			

### 3.2. *Toxocara canis* eggs retrieved in dog fecal and hair samples

Dogs from indigenous communities presented *T. canis* eggs in 9/194 (4.6%) fecal samples, of which 3/32 (9.4%) were from Paraná State, and 6/162 (3.7%) were from São Paulo (Table 4). Among the hair samples, *T. canis* eggs were retrieved in 4/204 (2.0%), of which 1/162 (0.6%) were from São Paulo (*n* = 27 eggs), and 3/42 (7.1%) were from Paraná (*n* = 7 eggs; range = 1–4 eggs). All the recovered eggs were classified as viable (nonembryonated).

**Table 4:** Frequency of *Toxocara* spp. eggs in fecal and hair samples of dogs living in indigenous communities of Paraná and São Paulo States, Brazil.

Communities	Samples: positive /total (%)		Feces	Hair
Paraná		
Araça’í	2/20 (10.0)	2/20 (10.0)
Guaviraty	1/12 (8.3)	0/12 (0.0)
Tupã Nhe’e Kretã	N.S.	1/10 (10.0)
Total	3/32 (9.4)	3/42 (7.1)
São Paulo		
Ekeruá	1/44 (2.3)	0/44 (0.0)
Kopenoty	5/50 (10.0)	1/50 (0.0)
Nimuendaju	0/35 (0.0)	0/35 (0.0)
Tereguá	0/33 (0.0)	0/33 (0.0)
Total	6/162 (3.7)	1/162 (0.6)

N.S., not sampled.

### 3.3. *Toxocara* spp. eggs in soil samples

A total of 130 soil samples were collected, of which 90 were from Paraná, and 40 were from São Paulo state. In Paraná, 36/90 (40.0%) soil samples were positive for *Toxocara* spp. (Table 5). Presence of *T. canis* eggs from soil was observed only in Paraná communities, most frequently in the Araça-i community (70.0%; average of eight eggs/50 g of soil), followed by Tupã Nhe’é Kretã (26.6%; average of one egg/50 g of soil) and Guaviraty (23.3%; average of one egg/50 g of soil). According to the classification criteria (Supplementary Figure S8), most retrieved *Toxocara* spp. eggs (56/121; 46.3%) had larvae (embryonated eggs) or were classified as viable (53/121; 43.8%). Approximately 10% of the retrieved eggs were classified as either embryonated (7/121; 5.8%) or non-viable (5/121; 4.1%). No *Toxocara* eggs were found in the 40 samples tested from the São Paulo community.

**Table 5:** *Toxocara* spp. eggs retrieved from soil samples collected in indigenous communities of Paraná (Southern) and São Paulo (South-eastern) States, Brazil, and morphological characteristics according to Roddie et al. (44) criteria.

Communities	Positive (%)	Morphological characteristics of *Toxocara* spp. eggs			
Paraná	V	NV	EM	E
Guaviraty	7/30 (23.3)	9	0	2	0
Araça’í	21/30 (70.0)	36	2	5	52
Tupã Nhe’e Kretã	8/30 (26.6)	6	3	0	4
	Total (%)	53/121 (43.8)	5/121 (4.1)	7/121 (5.8)	56/121 (46.3)

Communities	Positive (%)	Morphological characteristics of *Toxocara* spp. eggs			
São Paulo	V	NV	EM	E
Kopenoty	0/10	0	0	0	0
Tereguá	0/10	0	0	0	0
Ekeruá	0/10	0	0	0	0
Nimuendajú	0/10	0	0	0	0
	Total	0	0	0	0

V, viable; NV, non-viable; EM, embryonated egg; E, embryonated (containing larvae).

### 3.4. Molecular characterization (PCR)

Genetic amplification revealed *Toxocara canis* DNA in eggs retrieved from soil samples, all collected in the indigenous communities of Paraná State, including Araça-i, Guaviraty, and Tupã Nhe’é Kretã (Supplementary Figure S9). No *T. cati* DNA was identified in the analyzed samples.

## 4. Discussion

To the best of our knowledge, the present study is the first to assess the seroprevalence and associated risk factors for toxocariasis in indigenous populations using a One Health approach, including dogs, soil, and healthcare professionals. This study has shown high seropositivity to *Toxocara* spp. in Brazil and indigenous individuals in Brazil (342/463; 73.9%). The rates reported in the current study are slightly higher than the seropositivity reported in a serosurvey involving a rural adult population in southern Brazil (247/344; 71.8%) (26). Living in rural areas has previously been reported as a risk factor for *Toxocara* seropositivity (OR:1.8), corroborating the results of a global meta-analysis (22).

One of the factors that may influence the seroprevalence rate in a population concerns the technique used to antibodies detection. The indirect ELISA tests using TES antigens have been the most commonly employed tests to assess the epidemiological status of toxocariasis in human populations (47). The study herein was based on an ELISA (sensitivity 78.3%; specificity 92.3%) and used 96 negative samples to calculate the cut-off value plus three standard deviations, increasing the rigor for determining the test as positive. Further, pre-adsorption of each tested sera sample with *A. suum* adult worm extract was applied to mitigate cross-reactivity with other *Ascaridia* (36).

Toxocariasis seroprevalence in Latin American indigenous populations have been observed in children (1/7; 14.3%) and adults (4/43; 9.3%) Warao Venezuelan indigenous (48), and in Tepehuanos adults (33/126; 26.2%) in Mexico (49). In a study in Colombia, a high prevalence (383/483; 79.3%) was observed in Wiwa people as a consequence of indigenous lifestyle characteristics, including no access to clean drinking water (rivers and unprotected wells used as the water source) or sanitary installations, access of stray dogs and cats to villages and living areas, and consequent egg contamination of soil and vegetables, under favorable tropical climate for *Toxocara* spp. transmission (24). Some Brazilian indigenous populations are also exposed to these risk factors, such as poor sanitation, which may explain the 2.72-fold increased risk for seropositivity in indigenous people living in Paraná State, southern Brazil. In contrast, São Paulo State communities presented more access to sanitation conditions (such as artesian wells for water supply and septic tanks for feces disposal) and were found to have lower seroprevalence.

The logistic regression model revealed the water source as the only risk factor for toxocariasis, with an 11.4-fold higher likelihood of positivity in indigenous people that used the river as the primary drinking water source. Similarly, a study in Moscow suggested that water is a disseminating factor for *Toxocara* spp. eggs, which was related to cats and dogs accessing internal reservoirs used by humans swimming and accidentally swallowing contaminated water (50). In addition, the high frequency of *Toxocara* spp. eggs observed in the Paraná State communities may have indicated a waterborne transmission of toxocariasis in addition to the soil transmission, as rainfall may spread embryonated eggs present in soil into the local river. Finally, a meta-analysis review considered untreated or unfiltered water consumption as a risk factor for human toxocariasis (22), because filtration and sedimentation may be sufficient to remove *Toxocara* spp. embryonated eggs (51). Therefore, access to clean drinking water and adequate sanitary facilities may play an essential role in reducing and preventing toxocariasis in indigenous populations.

As previously observed in Brazil (26, 52) and Nigeria (53), absence of significant association between toxocariasis risk factors (gender, age, educational level, consumption of raw or undercooked meat, consumption of game meat, owning dogs or cats) herein may be related to high human seropositivity and consequent difficulties for statistical significances. Despite the high number (250/463; 54.0%) of indigenous individuals declaring the habit of game meat consumption, only 24 (5.2%) reported eating raw meat (16/24 seropositive). Thus, the sample size may have been a limitation in reaching statistical significance for some variables, but such analysis should be performed with caution because of the possibility of prevarication bias.

Other studies have reported that male gender is a potential risk factor for *Toxocara* spp. seropositivity, particularly in males that are agricultural laborers as they are in close contact with soil (22, 54). Although the primary food source of indigenous communities was agricultural subsistence, gender was not found to be statistically associated with seropositivity in the current study. In addition, the proportions of seropositive males (50.6%) and females (49.4%) were similar, supporting that toxocariasis exposure was most likely gender independent. Similar findings have been observed in the indigenous populations of Colombia (24) and Mexico (49). This may be explained by the different social structures within different communities. In addition to soil contact during labor-related activities, drinking untreated water was also found to be a significant risk factor (24). Therefore, it is clear that the risk of human *Toxocara* spp. infection is higher in communities with contaminated soil that use water sources that contain *Toxocara* spp. eggs (55). This finding could explain the high exposure of both genders, particularly in communities with inadequate food sanitation practices or that wash with contaminated water (24).

Seropositivity was not found to be influenced by age, corroborating the results from indigenous populations in Mexico (49). However, this finding differed from that in Colombian indigenous communities, where a higher prevalence was observed in adults than in adolescents (24). Conversely, in rural settlers of the Brazilian Amazon, age more than 14 years was considered a protective factor (OR: 0.46) (56). Age remains a controversial risk factor in human toxocariasis, as younger individuals may be more likely to be infected due to ingestion of eggs from contaminated soil or sand or contact with dogs and cats (22, 57), while older adults may be seropositive due to antibody persistence and cumulative *Toxocara* spp. exposure during their lifespan (24). The results here suggest that younger and older people are likely to be exposed to contaminated soil during recreational activities and agricultural labor. Further studies should be conducted to fully elucidate the impact of age on the disease cycle.

Owning a cat or dog was not associated with toxocariasis seropositivity in indigenous individuals, possibly due to the lack of fencing and the use of leashes, which resulted in the presence of stray dogs and cats throughout indigenous communities. One study reported that dog and cat contact was a significant risk factor for toxocariasis, with a statistical influence of dog (OR = 1.5) and cat (OR = 1.6) contact on *Toxocara* spp. seropositivity in the under-18 population (58). The presence of stray dogs was found to increase the exposure risk in different communities of indigenous Crees in Canada (59). In addition, aboriginal school children raising dogs in eastern (60) and northeastern (23) Taiwan were 1.8- and 3.8-fold more likely to be seropositive, respectively.

The presence of *Toxocara* spp. eggs in dog feces (4.6%) and hair (2.0%) samples were considered low (particularly in communities of Paraná State), with results within the expected range of *Toxocara* spp. egg positivity by fecal examination of 11.7% (1.8–48.9%) in southern Brazil and 11.2% (0.7–39.0%) in southeastern Brazil (61). However, a study in Paraná State found *Toxocara* spp. eggs in 12/115 (10.4%) dog feces and 22/104 (21.2%) dog hair samples (52), indicating a potential underestimation in the current study, likely because only adult dogs were sampled. Younger dogs may drastically decrease shedding after 12 weeks, and after 40 weeks, infected dogs will not display any signs because of an adaptive immune response (8). In contrast, the poor nutritional and sanitary conditions observed in the present study may have predisposed adult dogs to *T. canis* infection via contaminated soil. The role of fox and wild dog populations as a potential source of environmental contamination has been argued (62), and should be considered herein. The possibility of soil contamination by *Toxocara* spp. eggs shed by wild canids living within the indigenous territory may have contributed to the parasite cycle and should be further investigated.

Soil contamination was observed only in Paraná State communities (23.3 to 70.0%), with a high frequency of embryonated eggs (56/121; 46.3%). Molecular DNA analysis of *Toxocara* spp. eggs obtained from soil samples from common areas revealed only *T. canis* contamination, likely due to the high circulation of wandering dogs in these sets. In Poland higher number of *T. canis* eggs were recovered in rural areas, while *T. cati* in urban areas (63). The tendency of cats burying their feces (64) may have influenced the lack of *T. cati* DNA amplification herein. Such result should be analysed with caution since DNA amplification by PCR was performed with a pool of the recovered eggs in each community and may not exclude the contamination by *T. cati*. Therefore, further investigations involving soil sampling within the yards of indigenous households should be conducted during all seasons to elucidate the presence of *Toxocara* spp. eggs in peri-domiciliary areas.

Non-indigenous healthcare professionals, particularly in Paraná State communities (46/147, 31.30%), were 9.2-fold more likely to be seropositive than those who sporadically visited the indigenous communities, suggesting exposure to *Toxocara* spp. during their work-related activities and 5 days a week. Nevertheless, indigenous individuals showed significantly higher seroprevalence than all healthcare professionals, probably because of daily exposure to *Toxocara* spp. over time. Drinking water was not considered a risk factor for healthcare professionals, indicating the presence of other sources of infection. Male healthcare professionals were 2.3-fold more likely to be seropositive; the reasons for gender differences among healthcare professionals are still unclear, and further investigations of the different roles of males and females should be explored. Nevertheless, the high seroprevalence observed in healthcare professionals is a public health concern, and studies focusing on the impact of toxocariasis on healthcare professionals are warranted.

As a limitation, the present study sampled indigenous pet owners by convenience, as they needed to voluntarily visit the local health unit to participate. The statistical analyses of associated risk factors were based on questionnaire responses, which may represent the perceptions of volunteers but not the actual events and habits, particularly of their dogs. The present study has not surveyed healthcare professionals themselves, as they may not be so aware about toxocariasis and other tropical neglected diseases and should also be educated in terms of food safety and the need of frequent hands washing and other hygiene procedures. In addition, these professionals may not have the opportunity of washing hands frequently in these communities, or maybe the installations were not so hygienic, and water tanks may have lacked regular cleaning. Thus, further studies should focus on the personal knowledge and hygiene of healthcare professionals, as potential associated risk factor for toxocariasis and other soil and water-borne diseases. Finally, future studies should investigate the role of *Toxocara* spp. in causing clinical symptoms in seropositive people to pinpoint the clinical impact of toxocariasis in such communities. In addition, the in-house ELISA used herein, the most widely employed test for toxocariasis serosurveys, was designed for general antibody detection and was unable to differentiate between recent and chronic infections. Due to the difficulties in accessing the indigenous communities, and indigenous refusal in stool samplings (even with the help of local nurses), no stool examination of the indigenous population was performed to assess the co-infection by *Ascaris lumbricoides*, which could interfere in the ELISA results. Although western blot can be used to confirm positive ELISA findings to reduce false-positive results (47), the present study was limited to antibody detection by ELISA using pre-adsorption to mitigate cross-reactivity with *A. lumbricoides*. As limitations, no western blot was conducted to confirm positive ELISA findings and reduce false-positive results, as TES antigens applied to western blot assays would boost the study’s strength. However, serum samples were pre-adsorbed with *A. suum* adult worm extract to mitigate cross-reactivity with other Ascaridia, as previously established (36).

In addition, despite efforts, no cat feces were sampled in the present study. First, indigenous owners refused cat stress during catching and restrain. Second, with mostly free-range and feral cats, no environmental feces could be differentiated from dog feces. The limitation of visits also reduced the possibility of collecting a higher number of soil samples to determine the frequency of *Toxocara* spp. eggs in different sets of each community. Nonetheless, future studies should include cat feces samplings to fully establish the cat role on toxocariasis in these communities.

In summary, indigenous populations worldwide face disproportionately high rates of diseases related to their environment and animals. This study reported high seroprevalence of toxocariasis in different indigenous populations in southern and southeastern Brazil. Not surprisingly, the highest *Toxocara* spp. seroprevalence was observed in indigenous communities with poor sanitary conditions that used a local river as their drinking water source. Healthcare professionals who worked full-time in indigenous communities were almost 10-fold more likely to be seropositive than those who visited sporadically, suggesting exposure to *Toxocara* spp. during work-related activities and raising concerns for their health. In addition, this study found that seroprevalence was significantly higher in indigenous individuals than in healthcare professionals, likely due to *Toxocara* spp. exposure over time.

## Data availability statement

The original contributions presented in the study are included in the article/Supplementary Material, further inquiries can be directed to the corresponding author.

## Ethics statement

The studies involving humans were approved by the Committee on Ethics in Human Health of the Brazilian Ministry of Health (protocol 52039021.9.0000.0102) at the Federal University of Paraná. The studies were conducted in accordance with the local legislation and institutional requirements. Written informed consent for participation in this study was provided by the participants’ legal guardians/next of kin. The animal study was approved by the Committee on Ethics in the Use of Animals (protocol number 033/2021) at the Federal University of Paraná. The study was conducted in accordance with the local legislation and institutional requirements.

## Author contributions

VS, LK, and AB contributed to the conception and design of the study and wrote the first draft of the manuscript. FD, IF, JF, RS, and SL organized the database. RG performed the statistical analysis. VS, IF, RG, LB, SL, CB, AS, LK, and AB wrote the sections of the manuscript. All authors contributed to the manuscript revision, read, and approved the submitted version.

## Funding

The present research was funded through the Brazilian National Council for Scientific and Technological Development-CNPq (404687/2021–0 and 401302/2022–9).

## Conflict of interest

The authors declare that the research was conducted in the absence of any commercial or financial relationships that could be construed as a potential conflict of interest.

## Publisher’s note

All claims expressed in this article are solely those of the authors and do not necessarily represent those of their affiliated organizations, or those of the publisher, the editors and the reviewers. Any product that may be evaluated in this article, or claim that may be made by its manufacturer, is not guaranteed or endorsed by the publisher.
