# Supplementary material for: One health approach to toxocariasis in Brazilian indigenous populations, their dogs, and soil contamination
Source: Front Public Health. 2023 Sep 7;11:1220001. doi: 10.3389/fpubh.2023.1220001 (PMC10517057; doi:10.3389/fpubh.2023.1220001)
Supplement: Supplementary file 1 [file Table_1.DOCX]

Supplementary Material

**Supplementary Table 1.** Locations and coordinates of Indigenous Communities in Paraná and São Paulo, Brazil, including the total and sampled populations.

| **Indigenous Communities** | **Coordinates** | **Total** | **Sampled** | **%** | **Ethnicity** |
| --- | --- | --- | --- | --- | --- |
| **Paraná State - South** |  |  |  |  |  |
| Tekoa Pindoty | 25°31'10.34"S 48°28'06.78"O | 40 | 22 | 55.0 | Guarani |
|  |  |  |  |  |  |
| Kuaray haxa | 25°18'40.65"S  48°18'9.14"O | 25 | 18 | 72.0 | Guarani |
|  |  |  |  |  |  |
| Araça'í | 25°29'23.31"S  49° 0'11.57"O | 90 | 72 | 80.0 | Guarani, Kaingang |
|  |  |  |  |  |  |
| - Tupã Nhe'e Kretã | 25°36'56.21"S 48°56'13.05"O | 30 | 29 | 96.7 | Guarani, Kaingang |
|  |  |  |  |  |  |
| Guaviraty | 25°36'25.35"S 48°26'36.33"O | 39 | 21 | 53.8 | Guarani |
|  |  |  |  |  |  |
| **São Paulo State - Southeast** |  |  |  |  |  |
| Kopenoty | 22°15’58.20’’S 49°21’00.95’’O | 245 | 125 | 51.0 | Guarani, Kaingang, Terena |
|  |  |  |  |  |  |
| Tereguá | 22°15'55.31"S 49°20'53.34"O | 127 | 47 | 37.0 | Terena, Guarani |
|  |  |  |  |  |  |
| Ekeruá | 22°16’28.05’’S 49°22’21.95’’O | 159 | 56 | 35.2 | Guarani, Terena |
|  |  |  |  |  |  |
| Nimuendajú | 22°17’30.81’’S 49°22’41.03’’O | 100 | 73 | 73.0 | Guarani, Terena |
|  |  |  |  |  |  |

**Supplementary Table 2.** Soil sampling sets and number of samples collected per indigenous community in Paraná and São Paulo states.

| **Communities** | **Soil sampling sets** | | | | **Total** |
| --- | --- | --- | --- | --- | --- |
| **Paraná** | **School** | **Health center** | **Praying house** | **Trails** |  |
| Guaviraty | 10 | - | 10 | 10 | 30 |
| Araça'í | 10 | 10 | 10 | - | 30 |
| Tupã Nhe'e Kretã | 10 | - | 10 | 10 | 30 |
|  | | | |  |  |
| **Communities** | **Soil sampling sets** | | | | **Total** |
| **São Paulo** | **School** | **Health center** | **Praying house** | **Trails** |  |
| Kopenoty | - | 5 | 5 | - | 10 |
| Tereguá | 5 | **-** | **-** | 5 | 10 |
| Ekeruá | 5 | **-** | **-** | 5 | 10 |
| Nimuendajú | 5 | **-** | **-** | 5 | 10 |


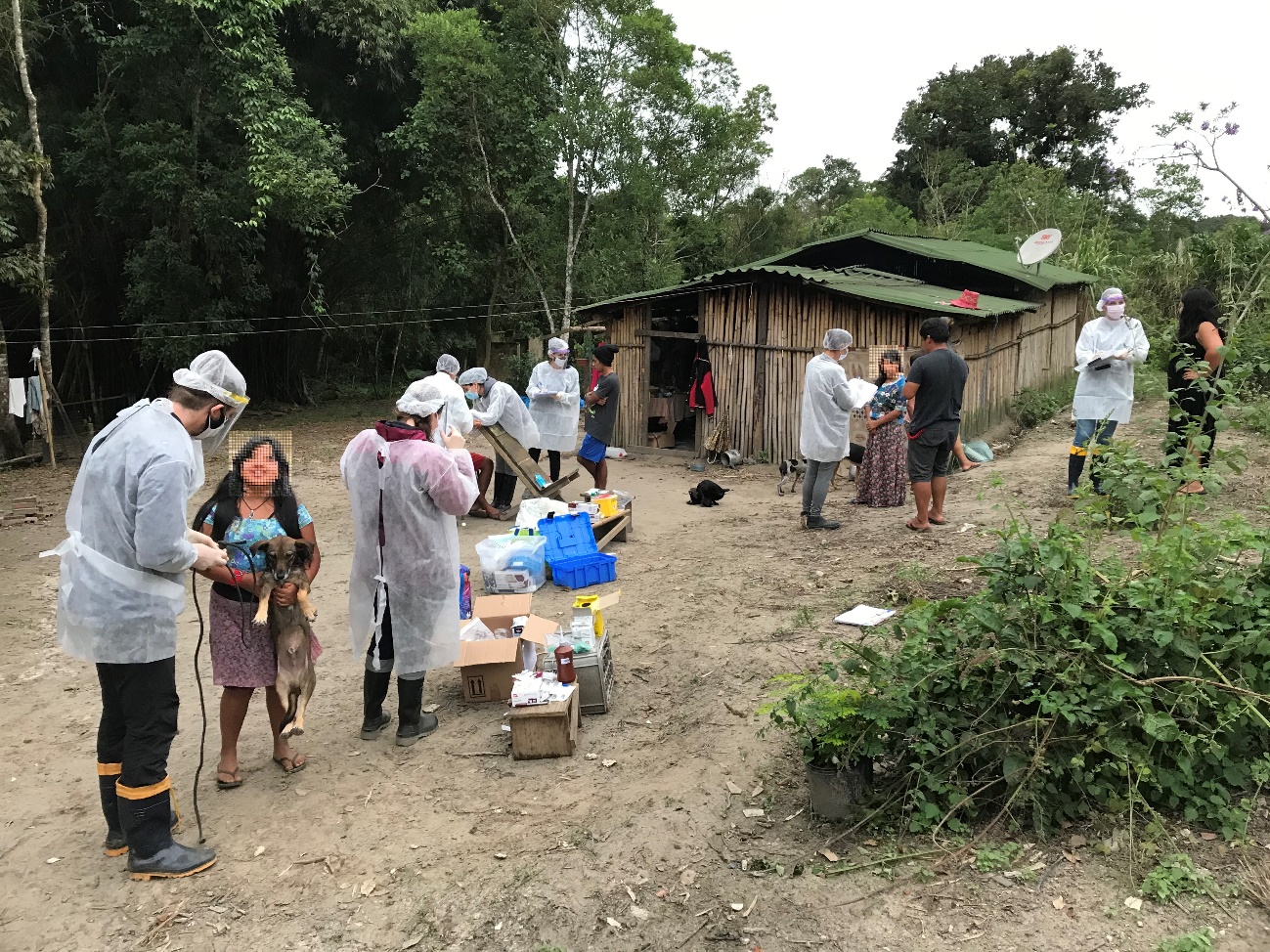


**Supplementary Figure 1.** Overview of the indigenous community of Tekoa Pindoty during samplings (with faces blurred), located on the oceanic island of Cotinga, Paranaguá City, Paraná State (photo: Alexander Welker Biondo).


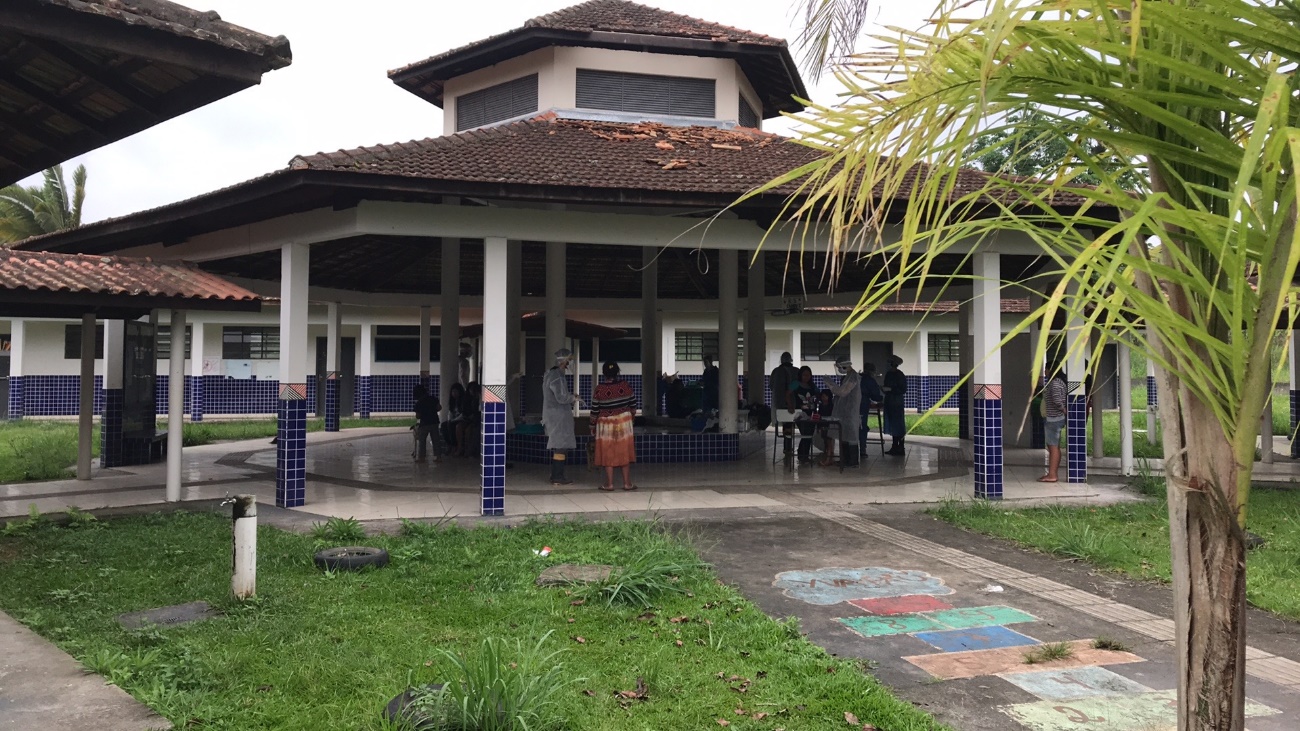


**Supplementary Figure 2.** Overview of the indigenous community of Kuray Haxa during samplings, located on seashore mainland, Guaraqueçaba City, Parana State (photo: Alexander Welker Biondo).


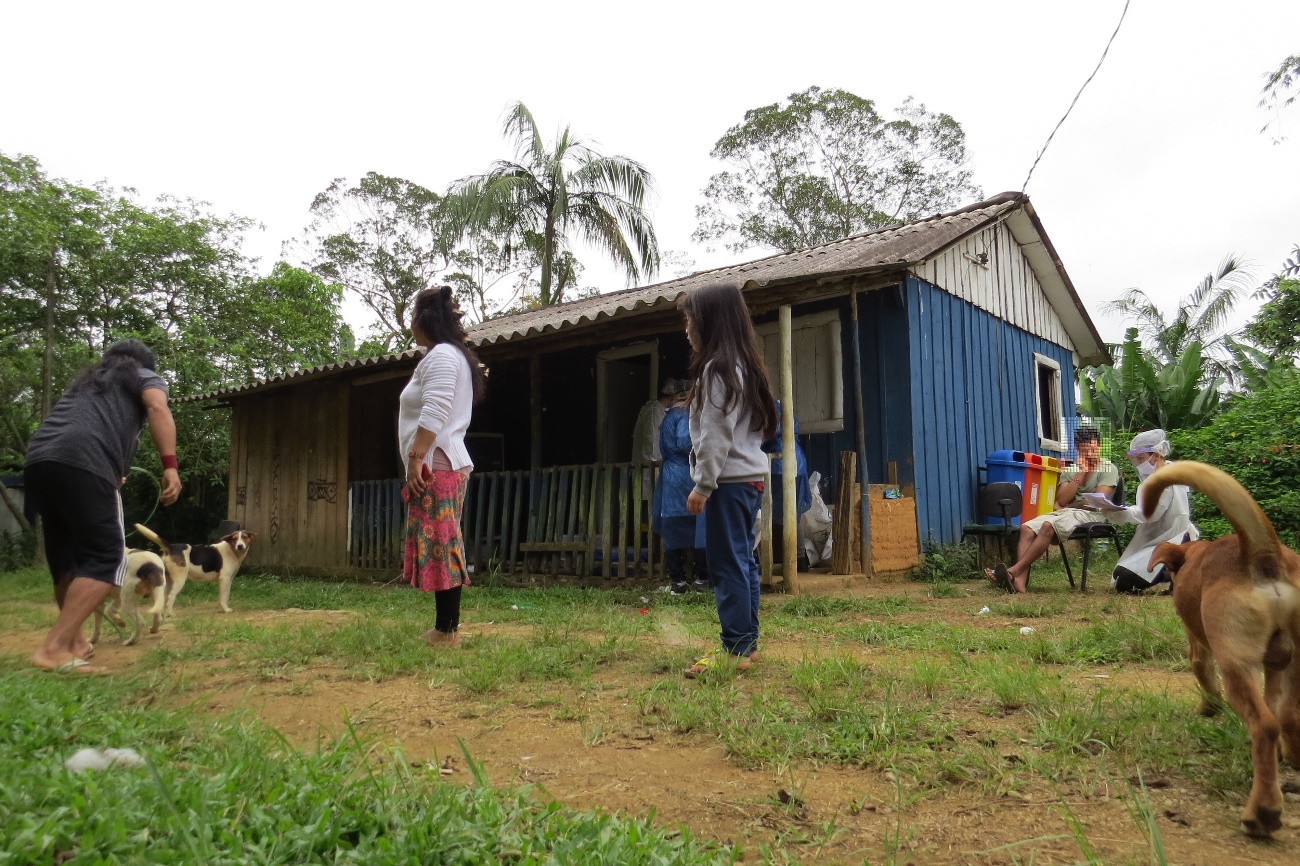


**Supplementary Figure 3.** Overview of the indigenous community of Guaviraty during samplings (with faces blurred), located on seashore mainland, Pontal do Paraná City, Parana State (photo: Alexander Welker Biondo).


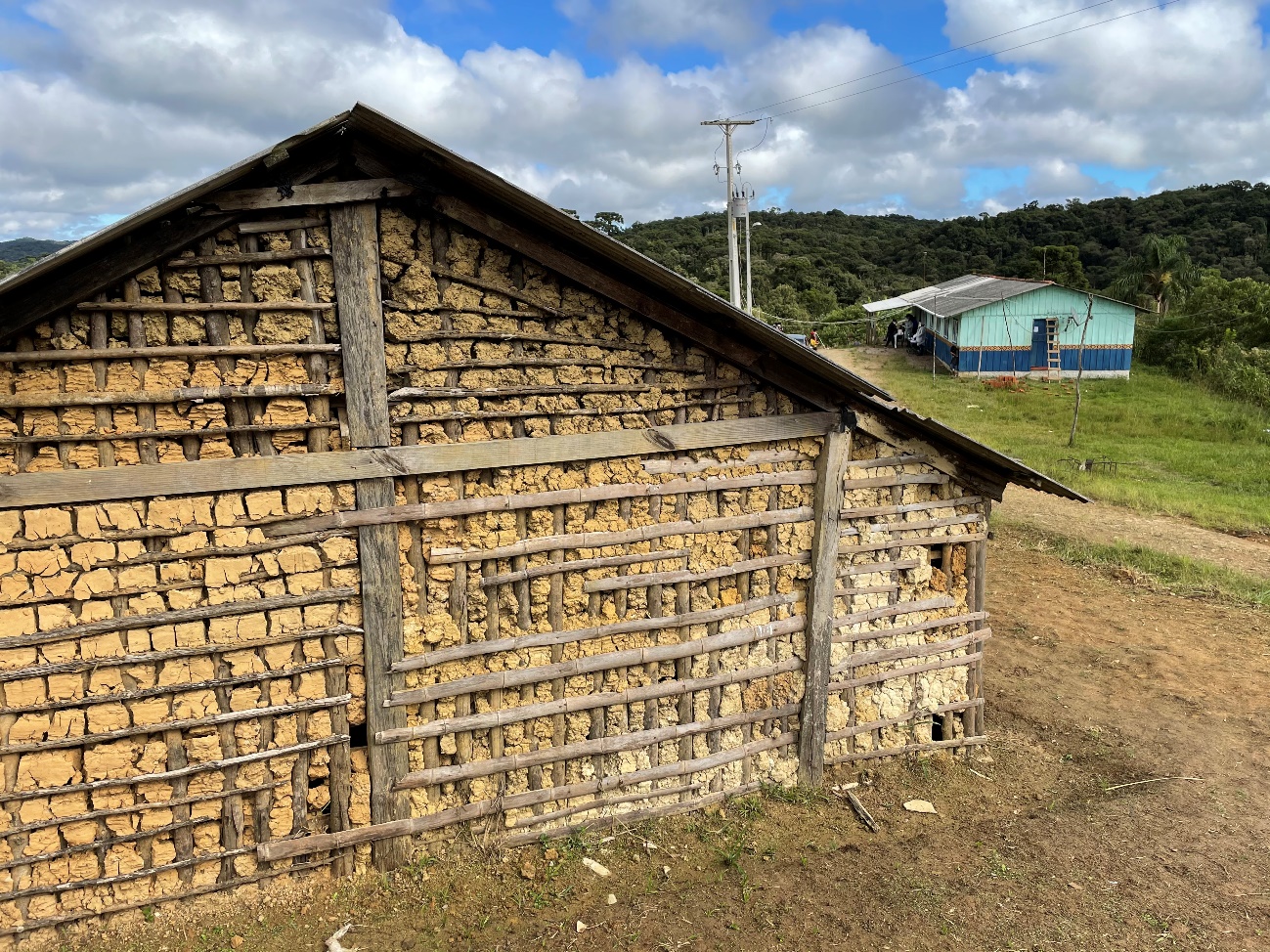


**Supplementary Figure 4.** Overview of the indigenous community of Tupã Nhe´é Kretã during samplings (car belonging to the research group), located on the Atlantic Forest Mountains, Morretes City, Parana State (photo: Alexander Welker Biondo).

**
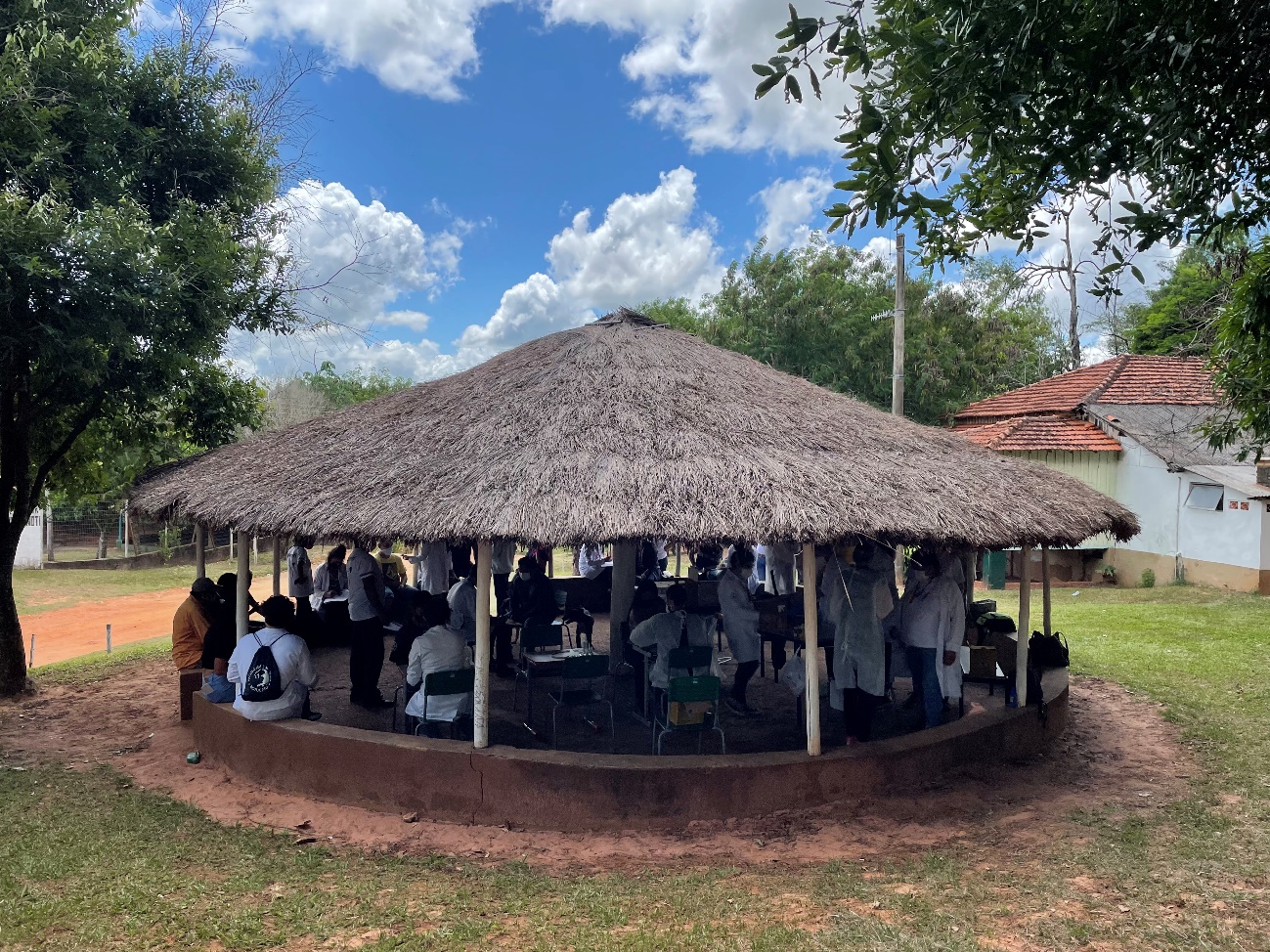
**

**Supplementary Figure 5.** Overview of the indigenous community of Kopenoty during samplings, located on countryside state, Bauru City, São Paulo State (photo: Alexander Welker Biondo).


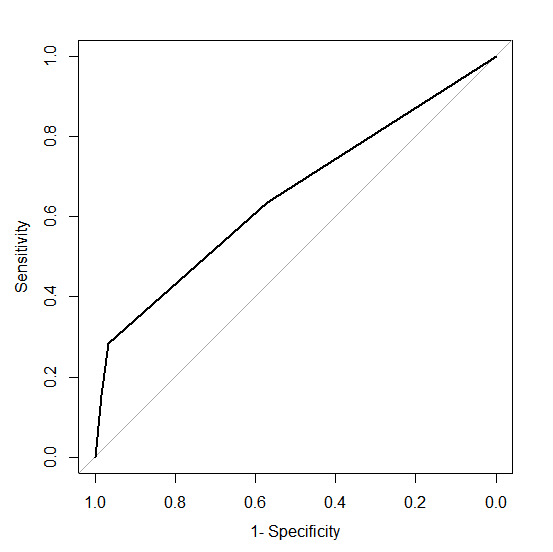


**Supplementary Figure 6.** Receiver operating characteristic (ROC) curve assessing the accuracy of the multivariate logistic regression model for predicting seropositivity for anti-*Toxocara* spp. antibodies in indigenous populations in indigenous communities of southern and southeastern Brazil (top; area under curve (AUC): 65.3%; 95% CI: 60.6-70.0).


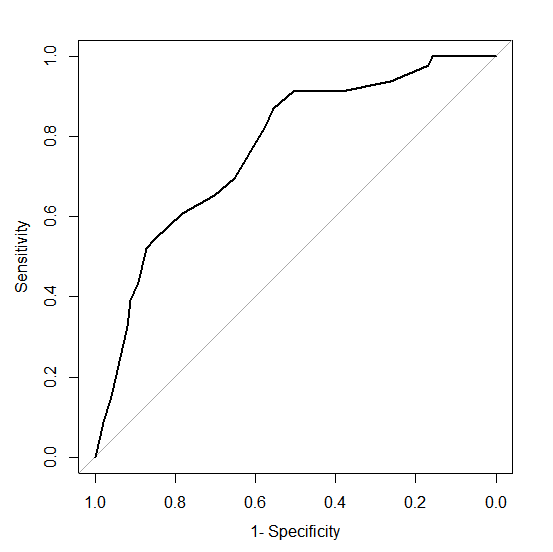
**Supplementary Figure 7.** Receiver operating characteristic (ROC) curve assessing the accuracy of the multivariate logistic regression model for predicting seropositivity for anti-*Toxocara* spp. antibodies in non-indigenous healthcare professionals of southern and southeastern Brazil (top; area under curve (AUC): 78.0%; 95% CI: 70.3-85.8).

##
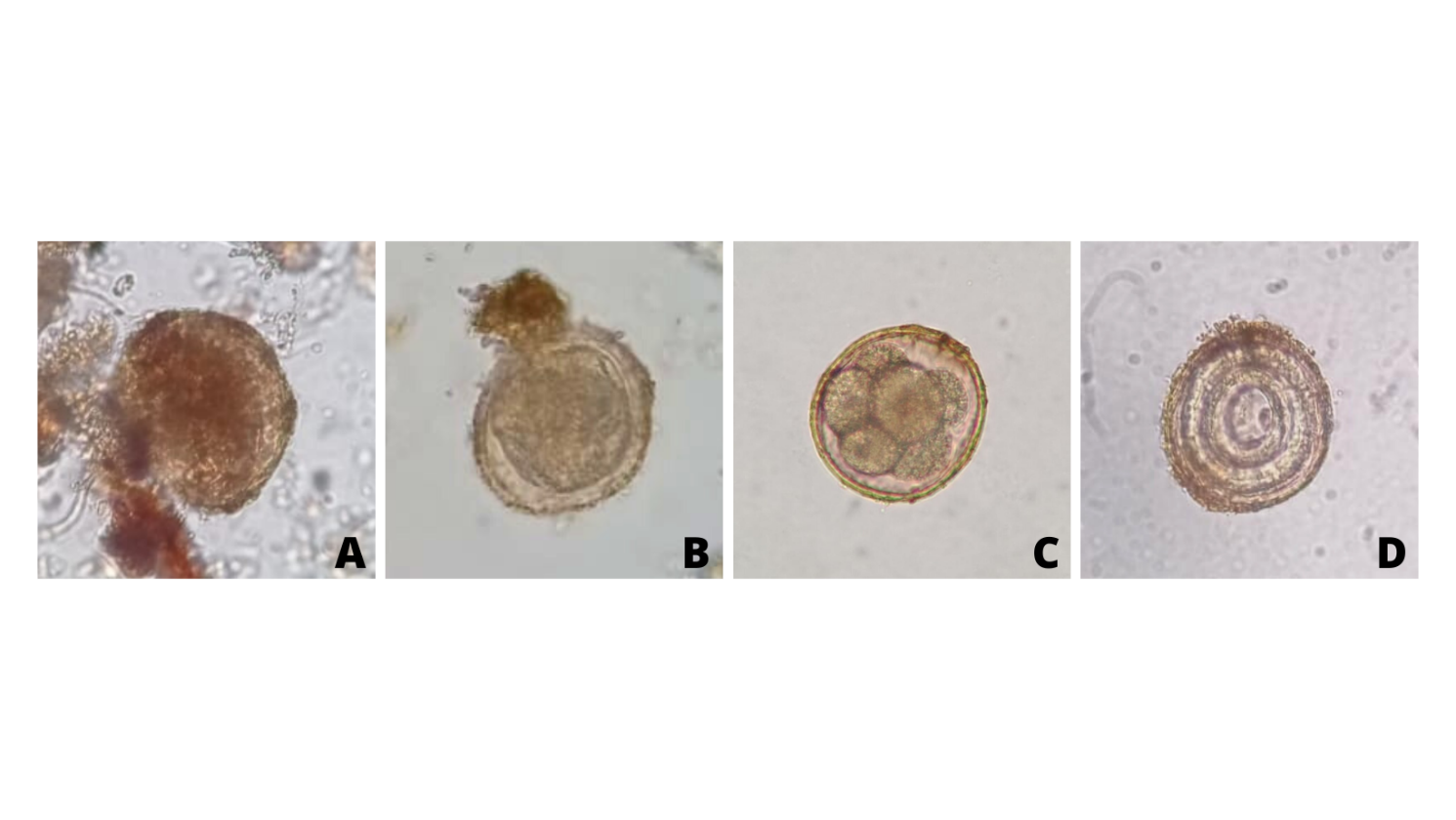


**Supplementary Figure 8.** Morphological aspects of *Toxocara* spp. eggs retrieved from soil samples collected in indigenous communities of Paraná and São Paulo States, Brazil, according to Roddie et al. (2008) criteria. (A) non-viable: egg not intact presenting wall disruption; (B) viable: intact egg with content; (C) embryonating egg: presenting cellular division; e (D) embryonated: egg containing larva. Magnification: 40x.

##
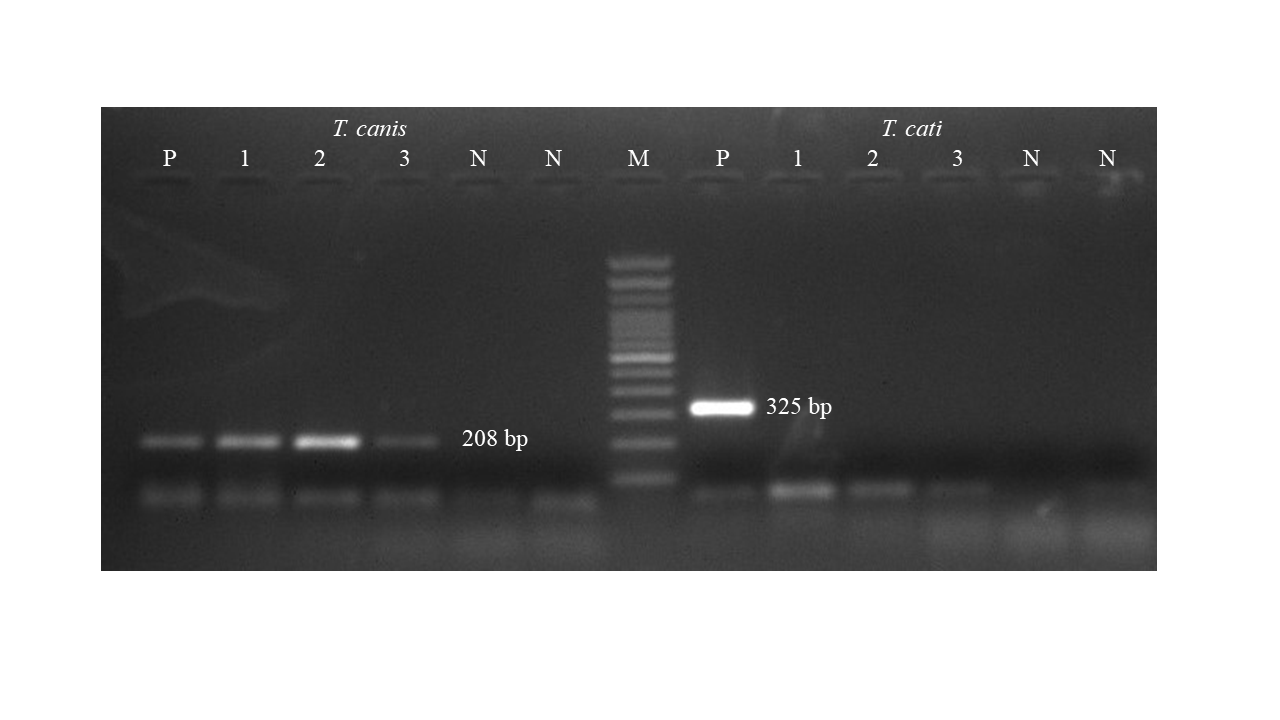


**Supplementary Figure 9.** Analysis of the polymerase chain reaction (electrophoresis on 1.5% agarose gel) amplified *Toxocara* spp. eggs recovered in soil samples from indigenous communities in Paraná State, Brazil, showing *T. canis* DNA amplification (208 bp). M: 100 bp DNA marker; P: Positive Control; N: Negative control; Tested sample by community: 1: Araça'í; 2: Guaviraty; 3: Tupã Nhe'é Kretã. No *T. cati* DNA (325 bp) was amplified in the tested samples.
